# The Cause of Severe Metabolic Acidosis With Vomiting in a Neonate

**DOI:** 10.7759/cureus.40973

**Published:** 2023-06-26

**Authors:** Aashka Patel, Shreya Sodhani, Louisdon Pierre, Adebayo Adeyinka, Noah Kondamudi

**Affiliations:** 1 Pediatrics, The Brooklyn Hospital Center, New York, USA; 2 Pediatric Critical Care Medicine, The Brooklyn Hospital Center, New York, USA

**Keywords:** failure to thrive, vomiting, severe dehydration, cow’s milk protein allergy, high anion gap metabolic acidosis

## Abstract

We present a 22-day-old male born full term who presented with worsening non-projectile, non-bilious vomiting and failure to thrive (FTT) and was admitted to the pediatric intensive care unit (PICU) for severe metabolic acidosis with an elevated anion gap. Despite changing the formula, the patient continued to have spit-ups after feeds since birth. Before this admission, his vomiting worsened with every feed, which was now forceful along with two days of loose stools. Obstructive causes of emesis were ruled out with an upper gastrointestinal series, and a decision was made to evaluate for organic causes of FTT. Transient resolution of symptoms was noticed when the patient was placed NPO (nothing by os/mouth) briefly. His symptoms returned on resuming cow milk-based formula feeds. At this time, a presumptive diagnosis of cow milk protein allergy (CMPA) was made. Positive fecal occult blood supported the diagnosis, and his formula was changed to an extensively hydrolyzed formula (eHF). This is a case of severe CMPA with prolonged vomiting and FTT presenting with severe metabolic acidosis with an elevated anion gap. This case report highlights how CMPA can lead to severe dehydration with metabolic acidosis and increased anion gap.

## Introduction

In everyday clinical practice, disorders related to the acid-base balance in the human body are commonly observed, highlighting the significance and complexity of this crucial physiological process. Metabolic acidosis occurs in many pathological processes, which increase blood hydrogen ion concentration or decrease bicarbonate ion concentration. The presence or absence of an elevated anion gap helps to delineate the etiology of metabolic acidosis [1]. Vomiting causes loss of hydrogen-rich gastric secretions and generally leads to metabolic alkalosis [2]. However, in our case, metabolic acidosis was found in a vomiting neonate. This is most likely due to the severity of the dehydration from persistent vomiting leading to a state of hypoperfusion and elevated lactate level. If the clinical presentation seems inconsistent with the expected acid-base disorder, it becomes imperative to further explore the underlying cause of the acid-base disorder.

## Case presentation

A 22-day-old male born full term presented to the emergency room (ER) with a two-day history of worsening non-bloody, non-bilious emesis and fussiness. The baby was noted to have had spit-ups since birth, and his formula was changed from regular Similac® to Gentlease® (both cow milk-based formulas), with no relief in his symptoms. Two days before the presentation, his emesis worsened and was now "forceful" after every feed, prompting the ER visit. The mother further reported that the patient's stools were looser in consistency than baseline and also endorsed a decrease in the number of wet diapers. In the ER, his physical exam demonstrated poor skin turgor, delayed capillary refill at four seconds, and sunken anterior fontanelle. The patient’s birth weight was 3260 g, whereas the weight on admission (day 22 of life) was 3201 g. As the patient had failed to regain birth weight, a decision was made to further investigate the cause of his poor weight gain and his acute presentation of vomiting.

**Figure 1:**
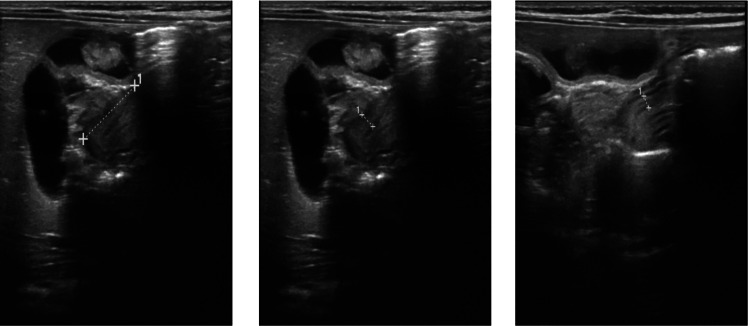
Ultrasound pylorus with a thickness of 3 mm, length of 13 mm, and diameter of 15 mm. Gastric emptying was noted with feeding.

His metabolic panel showed severe hyperchloremic metabolic acidosis with a bicarbonate level of 5, chloride of 121, and an elevated anion gap of 15. The patient was admitted to the pediatric intensive care unit (PICU) to manage severe metabolic acidosis and rule out surgical and non-surgical causes of the emesis with failure to thrive (FTT). Although his metabolic panel (Table 1) showed hyperchloremic metabolic acidosis which is not typical of pyloric stenosis, an abdominal ultrasound (Figure 1) was performed, which ruled out pyloric stenosis.

**Table 1:** Electrolyte trend throughout the hospital stay

	Day 1	Day 2	Day 3	Day 6	Prior to discharge
Sodium (mmol/L)	141	140	143	139	137
Chloride (mmol/L)	121	118	114	102	105
Bicarbonate (mmol/L)	5 (L)	7 (L)	11 (L)	24	21
Anion gap (mmol/L)	15 (H)	15 (H)	18 (H)	13	11

**Figure 2:**
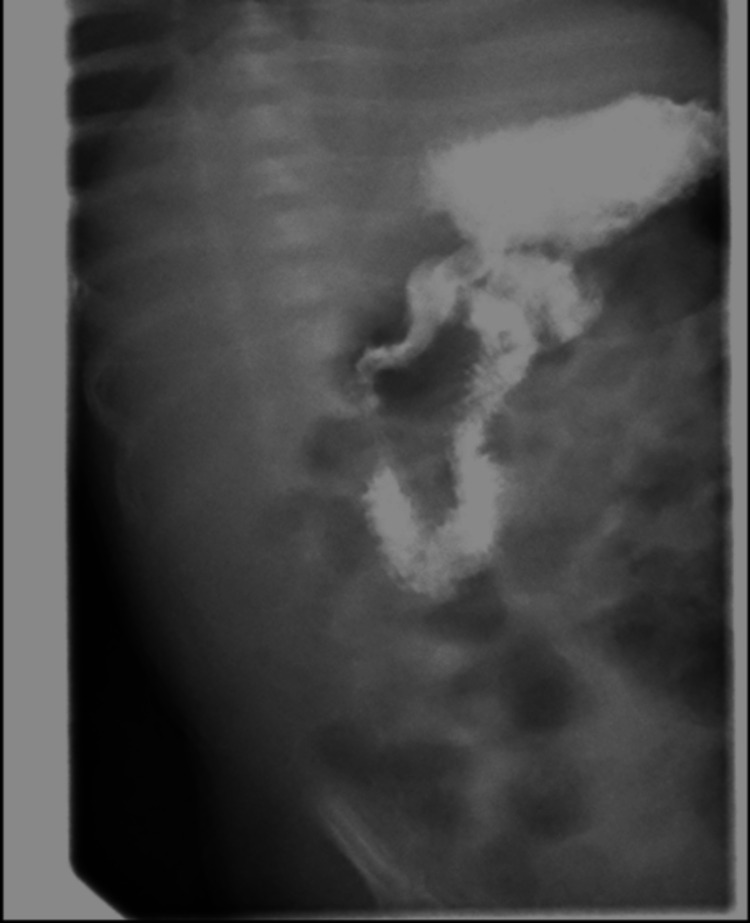
Upper gastrointestinal series showing no evidence of pyloric stenosis or other sites of obstruction

The patient was briefly placed on NPO for an upper gastrointestinal (GI) series (Figure 2), which ruled out other obstructive causes of emesis. While he was on NPO, there was an improvement in the consistency of his stools. However, when cow milk-based formula was resumed, the patient started to have loose stools and spit-ups again. The presumptive diagnosis of cow milk protein allergy (CMPA) was made at this time. The pediatric gastroenterology team was consulted, and his formula was switched to an extensively hydrolyzed formula (eHF) based on their recommendation. The fecal occult blood test (FOBT) was positive, further supporting our presumptive CMPA diagnosis. Improvement in his clinical symptoms with the resolution of metabolic acidosis (refer to Figure 1) confirmed our diagnosis, and he was discharged on eHF with outpatient gastroenterology follow-up.

## Discussion

**Figure 3:**
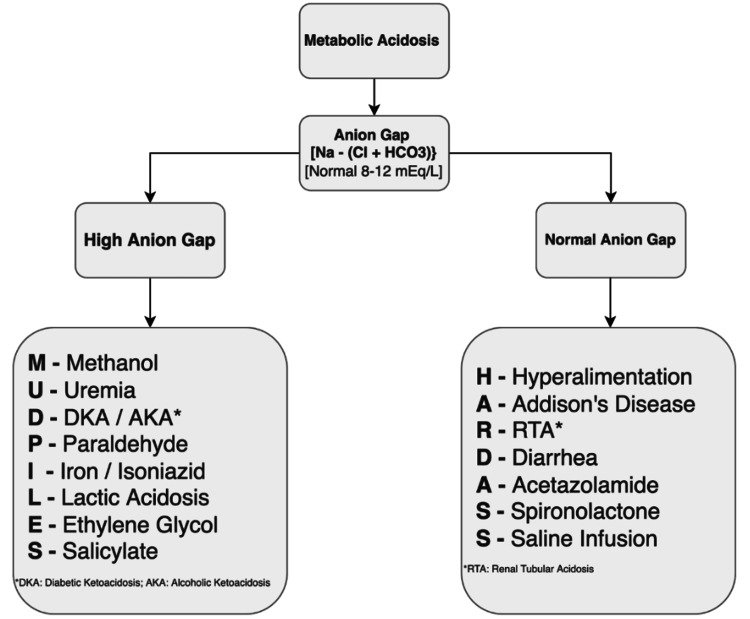
Etiologies of metabolic acidosis

Metabolic acidosis is defined as the reduction in serum bicarbonate levels (<22 mmol/L) or increase in serum hydrogen ion (H+) concentration that results in an arterial pH below 7.35 [3]. Causes of metabolic acidosis can be divided into two categories based on the presence or absence of a high anion gap (AG). AG is calculated from the serum sodium (Na), which is the principal cation in the blood, and chloride (Cl) and bicarbonate (HCO_3_), which are the principal anions (Na⁺ - [Cl⁻ + HCO_3_⁻]). High AG is defined when the difference is more than 12 mEq/L [4]. Etiologies of metabolic acidosis are illustrated in Figure 3 [5].

As our patient presented with severe non-bilious vomiting, we expected hypochloremic metabolic alkalosis from vomiting secondary to surgical or non-surgical causes. However, the opposite finding of hyperchloremic metabolic acidosis warranted further investigation. Using the above-mentioned algorithm, we concluded that the cause of our patient’s acid-base disturbance was lactic acidosis from severe dehydration and hypoperfusion. Lactic acidosis is of two types, namely, type A and type B. Type A lactic acidosis is due to hypoperfusion and hypoxia resulting in anaerobic glycolysis. This is seen in hypovolemic states like dehydration as well as all types of shock (hypovolemic, septic, cardiogenic, obstructive) and seizures [6]. Type B lactic acidosis is not associated with tissue hypoxia or hypoperfusion and occurs due to toxin-induced impairment of aerobic metabolism. Examples of type B lactic acidosis are ethanol intoxication, medications like metformin or epinephrine, malignancy, and mitochondrial dysfunction [7].

**Figure 4:**
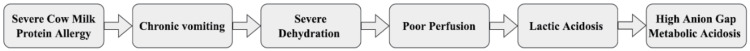
Severe CMPA causing high anion gap metabolic acidosis CMPA: Cow's milk protein allergy.

Our patient had signs of severe dehydration and hypoperfusion including sunken anterior fontanelle, delayed capillary refill, and poor skin turgor. The patient only had loose stools for two days, but his spit-ups and vomiting were significantly more of a chronic issue since birth. Chronicity of vomiting, which was now worsening, should have shown hypochloremic metabolic alkalosis. But in our case, the infant's vomiting was associated with an atypical acid-base disturbance of hyperchloremic metabolic acidosis with a high AG in contrast to what we expected. The elevated AG metabolic acidosis was due to lactic acidosis secondary to poor perfusion in the setting of severe dehydration as shown in Figure 4. After the exclusion of enteral feeding for a day or two, a diagnosis of CMPA was initially established, with improvement in the clinical symptoms. The severity of CMPA compromised the adequate feeding of the patient leading to poor weight gain of less than 3%.

CMPA is the most common food allergy in young children. The incidence of CMPA in developed countries is about 2%-6% with its highest prevalence being in the first year of life [8]. CMPA should be suspected in a child with a positive history of atopy [9]. Mild to moderate CMPA should be considered when infants present with immediate symptoms like spit-ups, vomiting, hives, angioedema, wheezing, or dry cough after consuming cow milk-based formula or late symptoms like eczema, diarrhea, blood in stools, iron deficiency anemia, or reflux [10-12]. Severe CMPA should be suspected if an infant presents with immediate severe symptoms like anaphylaxis and acute exacerbation of asthma or with late symptoms like chronic vomiting, chronic diarrhea, poor growth, iron deficiency anemia, or protein-losing enteropathy [10,11]. The gold standard for diagnosing CMPA is clinician-supervised oral food challenge when diagnosis with history is unclear [13-15].

## Conclusions

To conclude, CMPA can cause severe dehydration in patients, leading to tissue hypoperfusion, metabolic derangements, and a hypovolemic shock-like picture requiring aggressive fluid resuscitation. Metabolic derangements can be secondary to chronic underlying pathologies The aforementioned table and algorithm can aid in clinical diagnosis and management.
